# USP3 promotes gastric cancer progression and metastasis by deubiquitination-dependent COL9A3/COL6A5 stabilisation

**DOI:** 10.1038/s41419-021-04460-7

**Published:** 2021-12-20

**Authors:** Xiaosheng Wu, Hao Wang, Danping Zhu, Yixia Chai, Jing Wang, Weiyu Dai, Yizhi Xiao, Weimei Tang, Jiaying Li, Linjie Hong, Miaomiao Pei, Jieming Zhang, Zhizhao Lin, Jide Wang, Aimin Li, Side Liu

**Affiliations:** 1 Guangdong Provincial Key Laboratory of Gastroenterology, Department of Gastroenterology, Nanfang Hospital, Southern Medical University, Guangzhou, 510515 China; 2 Department of General Surgery, Nanfang Hospital, Southern Medical University, Guangzhou, 510515 China; 3 Guangdong Provincial Key Laboratory of Precision Medicine for Gastrointestinal Tumor, Guangzhou, 510515 China; 4 Department of Medical Examination, Rocket Army Guangzhou Special Service Convalescent Center, Guangzhou, 510515 China; 5 Department of Pathology, School of Basic Medical Sciences, Southern Medical University, Guangzhou, 510515 China; 6 Department of Gastroenterology, Longgang District People’s Hospital, Shenzhen, 518172 China

**Keywords:** Gastric cancer, Ubiquitylation

## Abstract

As an important regulator of intracellular protein degradation, the mechanism of the deubiquitinating enzyme family in tumour metastasis has received increasing attention. Our previous study revealed that USP3 promotes tumour progression and is highly expressed in gastric cancer (GC). Herein, we report two critical targets, COL9A3 and COL6A5, downstream of USP3, via the isobaric tags for relative and absolute quantification technique. Mechanistically, we observed that USP3 interacted with and stabilised COL9A3 and COL6A5 via deubiquitination in GC. Importantly, we found that COL9A3 and COL6A5 were essential mediators of USP3-modulated oncogenic activity in vitro and in vivo. Examination of clinical samples confirmed that elevated expression of USP3, concomitant with increased COL9A3 and COL6A5 abundance, correlates with human GC progression. These data suggest that USP3 promotes GC progression and metastasis by deubiquitinating COL9A3 and COL6A5. These findings identify a mechanism of GC metastasis regarding USP3-mediated deubiquitinating enzyme activity and suggest potential therapeutic targets for GC management.

## Introduction

Although the incidence of gastric cancer (GC) has declined in recent years, it remains the fifth most common malignancy and the fourth leading cause of cancer-related deaths worldwide [1]. Advances in surgical resection and chemotherapy have improved the prognosis of GC patients [2, 3]. However, a proportion of patients will eventually succumb to the disease as a result of metastasis due to missing the chance of curative surgery and thus leading to unsuccessful treatment in clinical settings [4]. Therefore, understanding the complex intrinsic mechanism of GC metastasis, mining promising molecular markers, and providing therapeutic targets for the early prevention, diagnosis, and treatment of GC metastasis is of great importance for improving the clinical outcome and prognosis of GC patients.

Ubiquitination is a posttranslational modification that plays critical roles in a diverse array of cellular processes [5, 6]. The ubiquitination of proteins is not a one-way process and thus can be reversed by a class of proteases known as deubiquitinating enzymes (DUBs), which catalyse the breaking of the isopeptide bond between the C-terminal glycine of ubiquitin (Ub) and the ε-amino group of lysine residues in target proteins [7, 8]. There are ~100 DUBs in the human proteome belonging to six families: ubiquitin-specific proteases, ubiquitin carboxyl-terminal hydrolases, ovarian tumour proteases, Machado-Joseph disease protein domain proteases, JAMM/MPN domain-associated metallopeptidases, motif-interacting with ubiquitin-containing novel DUB family, zinc finger with UFM1-specific peptidase domain protein, and monocyte chemotactic protein-induced protein [9]. Ubiquitin-specific protease 3 (USP3), a member of the USP family, is highly expressed in a variety of malignancies and is associated with a series of malignant biological behaviours of cancers [10–12]. USP3 has served as a deubiquitinase of Cdc25A and accelerated tumour proliferation, resulting in poor prognosis in patients with breast cancer [13]. Our previous work has demonstrated that in GC, USP3 expression is abnormally high in tumours and promotes epithelial-to-mesenchymal transition (EMT), invasion, and metastasis of cancer cells by deubiquitinating SUZ12 [14]. Results from other studies also support the conclusion that overexpression of USP3 increases tumour proliferation and is associated with the progression of GC [15]. Although several studies have reported the altered expression and preliminary functions of USP3 in GC, existing findings regarding the driver role of USP3 and its detailed downstream targets in GC are limited; therefore, further studies are required to provide more evidence supporting USP3 as an essential oncogene in GC and further insights on the complex regulatory network of USP3-mediated deubiquitination.

To further explore the potential roles of USP3 and its downstream mechanisms, we systematically detected the underlying proteins whose post-transcriptional modification might be involved in USP3-mediated deubiquitination by utilising the isobaric tags for relative and absolute quantification (iTRAQ) technique. Herein, we report two critical targets of USP3, COL9A3 and COL6A5, and the specific mechanism of USP3-mediated deubiquitination of COL9A3 and COL6A5. Moreover, we analyse the effects of such a posttranscriptional regulation axis on tumour cell EMT, invasion, and migration in GC. As members of the collagen family, COL9A3 and COL6A5 serve as essential skeletons in the extracellular matrix (ECM), which is the major component of the tumour microenvironment [16, 17]. However, there are no reports on the detailed regulatory mechanism of the two proteins from the perspective of posttranslational modification and their effects on malignant biological processes, including EMT, in GC.

In this study, we aimed to present and validate a molecular mechanism by which USP3 deubiquitinates and stabilises COL9A3 and COL6A5 to promote cell EMT, invasion, and migration in GC. Our findings help to elucidate the mechanism landscape for USP3 regarding its roles in regulating GC progression and supporting molecules, including USP3, COL9A3 and COL6A5 as the underlying targets for GC management in clinical settings.

## Results

### USP3 maintains COL9A3 and COL6A5 stability

**Fig. 1:**
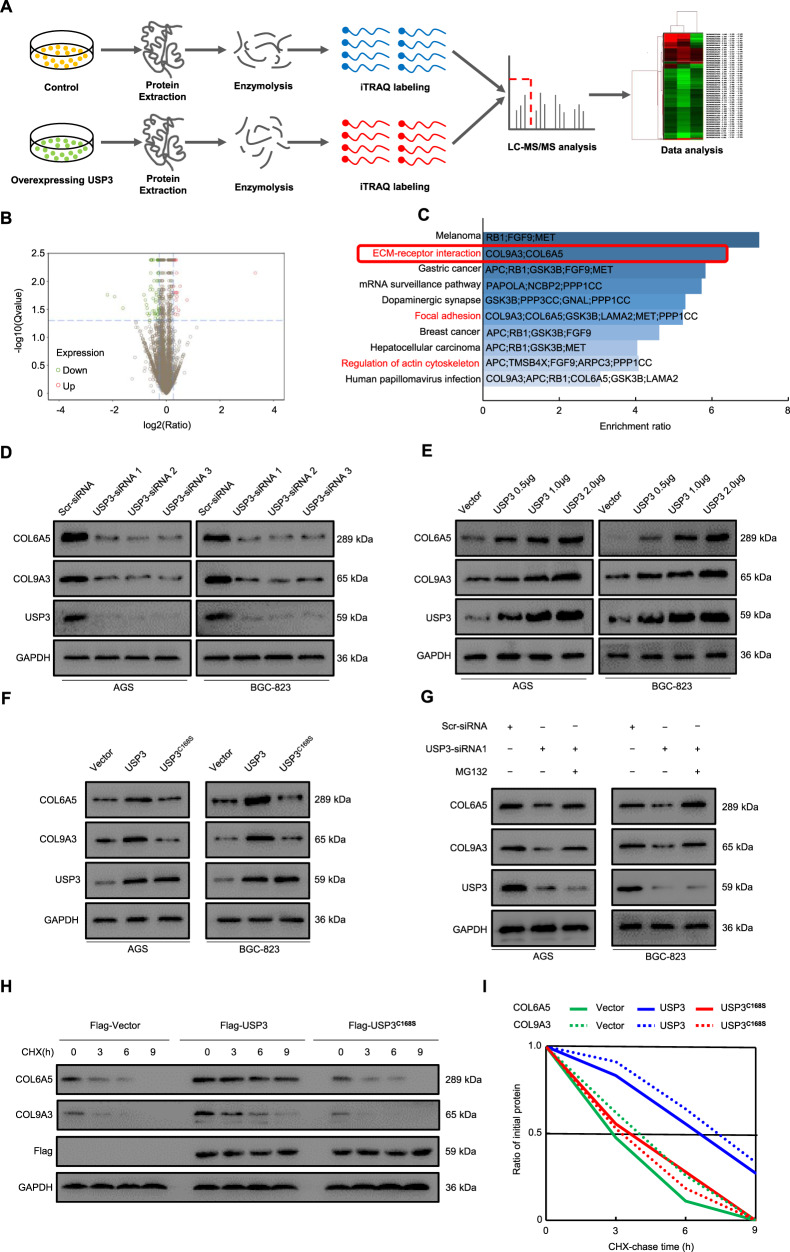
USP3 maintains COL9A3 and COL6A5 stability. **A** Framework of the iTRAQ labelling quantitative proteomic study. **B** Volcano plot showing differentially expressed proteins. **C** Significant KEGG pathways associated with USP3 expression. **D** Knockdown of USP3, by transfecting cells with three independent siRNAs for 48 h. The expression of USP3, COL9A3 and COL6A5 was detected by western blot. **E** Western blot detected COL9A3 and COL6A5 expression in GC cells, after transfecting different volumes of USP3-overexpressed plasmids. **F** COL9A3 and COL6A5 levels in AGS and BGC-823 cells after introducing USP3 or C168S. **G** GC cells transfected with the indicated siRNA were treated with or without the proteasome inhibitor MG132 (20 μM, 8 h), and then proteins were analysed. **H** AGS and BGC-823 cells transfected with the indicated plasmids were treated with cycloheximide (10 μg ml^−1^), and collected at the indicated times for western blot. **I** Time-dependent variations of COL9A3 and COL6A5 levels are shown.

To identify differentially expressed proteins that may serve as potential interaction targets of USP3, we initially performed iTRAQ experiments to systematically detect the expression changes in the protein profile of the USP3-overexpressed GC cell line BGC-823, compared with the control (Fig. 1A). Among detected 5631 proteins, 122 (including 63 downregulated and 59 upregulated) were identified with significant expression alterations (Fig. 1B). KEGG pathway enrichment analysis was performed for the differentially expressed proteins to explore the biological function (see “Supplementary Materials and Methods” for details). Ten KEGG pathways were significantly enriched, as shown in Fig. 1C. In a previous study, we demonstrated that USP3 promotes metastasis by enhancing EMT in GC [14]. Consistently, above bioinformatics analyses revealed several canonical EMT-associated pathways that were significantly enriched, including “ECM–receptor interaction”, “focal adhesion” and “regulation of actin cytoskeleton” [18–20]. In particular, the ECM-receptor interaction pathway yielded the highest enrichment ratio among all the EMT-associated pathways that involved two collagen proteins, COL9A3 and COL6A5, which were significantly upregulated when USP3 was overexpressed in the iTRAQ experiments. Therefore, we focused on the expression association and interactions between USP3 and the above two proteins in the following analyses.

Western blot analysis showed that depletion of USP3 by three independent siRNAs markedly decreased COL9A3 and COL6A5 protein levels in AGS and BGC-823 cells (Fig. 1D). In addition, upregulation of USP3 protein expression resulted in the elevation of COL9A3 and COL6A5 proteins in a dose-dependent manner (Fig. 1E). However, real-time quantitative reverse transcription (qRT)-PCR analysis showed that neither knockdown nor overexpression of USP3 had effects on the mRNA levels of COL9A3 and COL6A5, which indicated that USP3 regulated the expression of COL9A3 and COL6A5 at the post-transcriptional level (Supplementary Fig. 1A). Interestingly, we found that USP3 failed to induce COL9A3 and COL6A5 expression after inactive mutation of the active site (C168S) for its DUB, suggesting that the regulatory effect of USP3 on COL9A3 and COL6A5 was dependent on its DUB activity (Fig. 1F). Moreover, MG132, a potent inhibitor of the 26S proteasome which catalyses the degradation of ubiquitinated proteins, could reverse the decrease in COL9A3 and COL6A5 proteins caused by USP3 siRNA treatment, indicating that USP3 might block the ubiquitin-mediated degradation of COL9A3 and COL6A5 (Fig. 1G). To address whether and how USP3 stabilised COL9A3 and COL6A5, we treated GC cells with the protein synthesis inhibitor CHX and examined the protein levels after different treatment durations. The results showed that the half-life of COL9A3 and COL6A5 was prolonged in cells overexpressing USP3 but shortened in cells with depletion of USP3 or its DUB (Fig. 1H, I and Supplementary Fig. 1B). These findings suggest that USP3 increases COL9A3 and COL6A5 protein levels through DUB activity to enhance their stability.

### USP3 interacts with and deubiquitylates COL9A3 and COL6A5

**Fig. 2:**
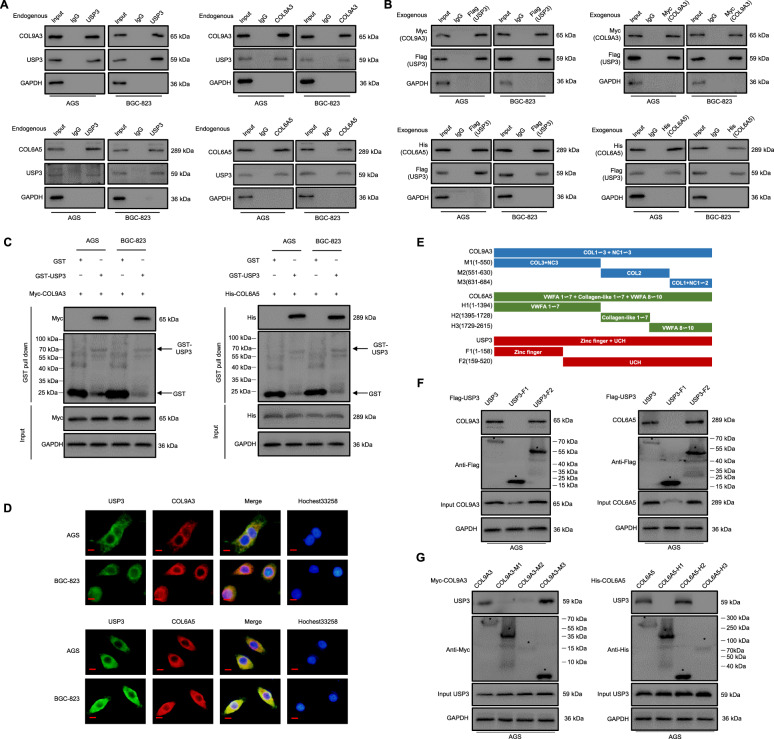
USP3 interacts with COL9A3 and COL6A5. **A** Cell lysates were subject to immunoprecipitation with control IgG, anti-USP3, COL9A3 or COL6A5 antibodies. Western blot detected the immunoprecipitates. **B** AGS and BGC-823 cells transfected with the indicated plasmids were subject to immunoprecipitation with anti-Flag, Myc or His antibodies. The lysates and immunoprecipitates were analysed. **C** AGS and BGC-823 cells transfected with COL9A3 or COL6A5 were lysed and lysates incubated with GST or GST-USP3. Proteins retained on sepharose were blotted with the indicated antibodies. **D** Double staining for USP3 and COL9A3/COL6A5 in GC cells, as visualised by confocal microscopy, with the nuclei counterstained with Hoechst 33258. Scale bars, 20 μm. **E** Overview of USP3, COL9A3 and COL6A5 structures. **F**, **G** AGS cells transfected with the indicated constructs were subject to immunoprecipitation with anti-Flag, anti-Myc and anti-His antibodies. The lysates and immunoprecipitates were then blotted.

To elucidate the mechanisms responsible for the effects of USP3 in maintaining the stability of COL9A3 and COL6A5, we performed reciprocal Co-IP experiments using endogenous protein. The results showed that USP3 could interact with COL9A3 and COL6A5 (Fig. 2A). Furthermore, exogenous Flag-tagged USP3, Myc-tagged COL9A3, and His-tagged COL6A5 constructs were co-transfected into GC cells. We found that Flag-tagged USP3 co-immunoprecipitated with Myc-tagged COL9A3 and His-tagged COL6A5 (Fig. 2B). In addition, GST pull-down assays were performed to validate the in vitro results showing that GST-USP3 effectively decreased COL9A3 and COL6A5 recombinant proteins in AGS cells. Similar results were observed for BGC-823 cells (Fig. 2C). Furthermore, the localisation of USP3, COL9A3, and COL6A5 was visualised using confocal laser scanning microscopy, in which the proteins were observed with marked colocalization in GC cells (Fig. 2D). The above results consistently support the conclusion that USP3 interacts with COL9A3 and COL6A5 in GC cells. To further explore the effective domain of USP3, which acted with COL9A3 and COL6A5, we constructed segmented plasmids for two known domains (i.e., ZnF and UCH) of USP3, namely USP3-F1 and USP3-F2 (Fig. 2E). Co-IP analyses revealed that the UCH domain of USP3 mediated its interaction with COL9A3 and COL6A5, while the ZnF domain showed no combined effects with COL9A3 and COL6A5 (Fig. 2F). To investigate the exact regions for COL9A3 and COL6A5 that USP3 acted with and targeted, we generated three truncated fragments spanning full-length for COL9A3 and COL6A5 (Fig. 2E). Our experiments revealed that COL9A3 combined with USP3 by its M3 region, and COL6A5 interacted with USP3 through its H2 region (Fig. 2G). In summary, these findings suggest that USP3 physically interacts with COL9A3 and COL6A5. We then explored the biochemical effects of such physical interactions.

**Fig. 3:**
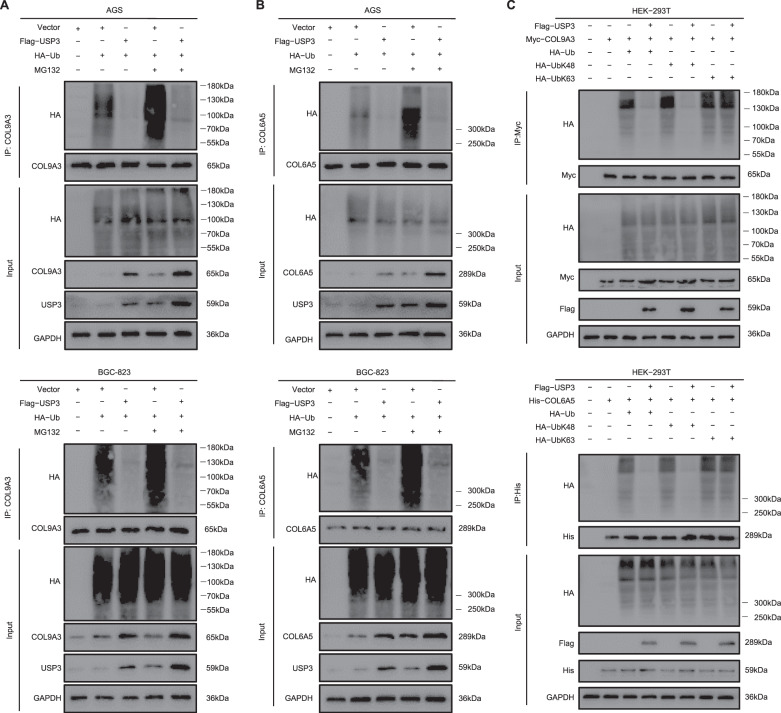
USP3 deubiquitylates COL9A3 and COL6A5. **A**, **B** HA-ubiquitin was coexpressed with Flag-USP3 in AGS cells. Western blot detected the ubiquitination of COL9A3 or COL6A5. **C** COL9A3 or COL6A5 ubiquitination linkage was analysed by western blot in HEK293T cells transfected with USP3, COL9A3 and COL6A5, and the indicated ubiquitin plasmids.

The deubiquitination effect of USP3 on COL9A3 and COL6A5 was examined in subsequent experiments. We transfected the ubiquitin-overexpressed plasmid into AGS and BGC-823 cells with or without USP3 overexpression and detected the ubiquitination levels of COL9A3 and COL6A5. Western blotting showed that the ubiquitination of COL9A3 or COL6A5 was strongly inhibited by USP3 overexpression. Furthermore, these findings were also observed consistently in the presence or absence of MG132, a potent inhibitor of the 26S proteasome, which catalysed the degradation of protein after ubiquitination modification (Fig. 3A, B). Consequently, we investigated which type of ubiquitination modification of COL9A3 and COL6A5 was removed by USP3. We examined the effect of USP3 on two universal types of ubiquitination, including Lys 48- and 63-linked ubiquitination. The results showed that USP3 effectively removed Lys 48-linked ubiquitination, but not the Lys 63-linked ubiquitination of COL9A3 and COL6A5 (Fig. 3C). These findings demonstrate that USP3, as a specific DUB, can interact with and deubiquitylate COL9A3 and COL6A5, increasing their stabilisation and protein levels.

### USP3 is involved in the EMT process to promote GC cell invasion and migration through COL9A3 and COL6A5

**Fig. 4:**
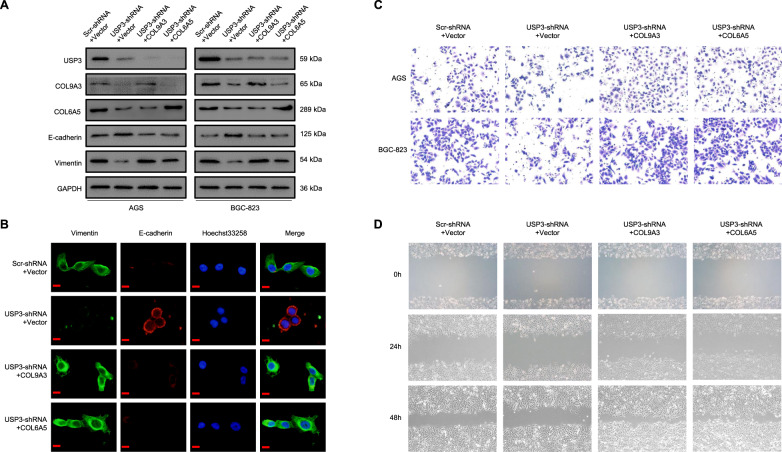
USP3 upregulates COL9A3 and COL6A5 to promote GC cells EMT, invasion and migration in vitro. COL9A3 or COL6A5 was introduced into the USP3 shRNA-transduced AGS and BGC-823 cells. **A**, **B** E-cadherin and Vimentin were analysed by western blot and immunofluorescence. Scale bars, 20 μm. **C, D** GC cell invasion and migration assays were performed.

The acquisition of the EMT phenotype is a critical process for switching early stage carcinomas into invasive malignancies, which is often associated with the loss of epithelial differentiation and gain of mesenchymal phenotype [21]. Previously, we found that overexpressed USP3 promoted GC cell EMT, invasion and migration [14]. Herein, we further evaluated the effects of COL9A3 and COL6A5 upregulation caused by USP3-mediated deubiquitination of USP3-induced GC cell EMT, invasion and migration. We constructed four groups, including the control (Scr-shRNA + Vector), USP3-knockdown (USP3-shRNA + Vector) and USP3-knockdown with COL9A3- or COL6A5-overexpressed (USP3-shRNA + COL9A3/COL6A5) groups, for subsequent cell experiments. Western blot and immunofluorescence assays in AGS and BGC-823 cells showed that depletion of USP3 increased the expression of E-cadherin and decreased vimentin expression, indicating the occurrence of EMT suppression, whereas the overexpression of COL9A3 or COL6A5 rescued the EMT ability in USP3-knockdown cells (Fig. 4A, B). We then performed transwell and wound-healing experiments to detect cell invasion and migration. The results showed that downregulation of USP3 reduced the ability of AGS cells to invade and migrate, while overexpression of COL9A3 or COL6A5 recovered the invasion and migration abilities of USP3-knockdown cells. Consistent phenomena were observed in BGC-823 cells (Fig. 4C, D and Supplementary Fig. 2A, B). On the contrary, USP3 overexpression upregulated vimentin but downregulated E-cadherin, indicating EMT phenotype promotion, while COL9A3 or COL6A5 knockdown inhibited cell EMT in the USP3-overexpressed group (Supplementary Fig. 3A, B). Transwell and wound-healing experiments revealed that downregulation of COL9A3 or COL6A5 distinctly inhibited the invasion and migration abilities of USP3-overexpressed GC cells (Supplemental Fig. 2C, D and Supplementary Fig. 3C, D). After overexpression of USP3 in COL9A3 or COL6A5 knocked down cells, the cell invasion and migration abilities could not be completely reconstituted (Supplementary Fig. 4).

**Fig. 5:**
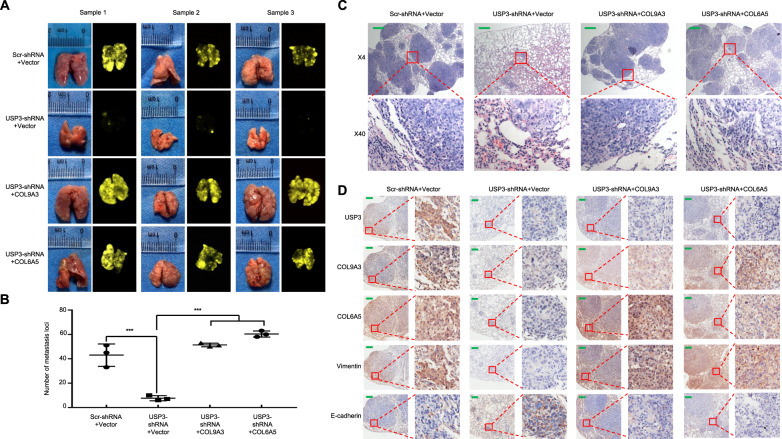
USP3 upregulates COL9A3 and COL6A5 to promote GC cells EMT, invasion and migration in vivo. **A** Tail vein injection with GC cells was performed in nude mice (*n* = 3 in each group). Images of metastatic loci in the lungs are shown. **B** The number of metastatic loci in the lungs was counted. **C** Metastatic cancer tissues were analysed by HE staining. **D** Vimentin and E-cadherin expression in the lung metastasis of GC was detected by IHC. The error bars represent the mean ± SD from three independent experiments. Scale bars, 200 μm in **C** and **D**.

To further investigate the effects of the USP3-COL9A3/COL6A5 axis on tumour metastasis in GC in vivo, we established BGC-823 cells stably depleting USP3 by lentiviral transfection, with simultaneous overexpression of COL9A3 or COL6A5. Tail vein injection with GC cells was performed to establish a lung metastasis model in nude mice, and four groups were analysed, including the control (Scr-shRNA + Vector), USP3 depletion (USP3-shRNA + Vector), and USP3 depletion with COL9A3 or COL6A5 overexpression (USP3-shRNA + COL9A3/COL6A5). On day 34 after the injection, the mice were sacrificed, lungs were collected, and metastatic colonies were counted (Fig. 5A, B). The presence of GC metastases in the lungs was confirmed by haematoxylin and eosin (HE) staining (Fig. 5C). We observed that the mice injected with USP3-knockdown GC cells had significantly less metastatic nodules in the lungs than those of the control (7.67 ± 1.20 vs 43 ± 5.29; *P* = 0.003). However, after COL9A3 (51.33 ± 0.88) or COL6A5 (60.33 ± 1.45) overexpression, the USP3-knockdown BGC-823 cells showed increased number of lung metastasis nodules (*P* < 0.001 for both, compared with the control) and rescued to a level similar to that of GC cell without USP3-knockdown (Fig. 5A, B). We further used IHC staining to analyse the expression of vimentin and its association with USP3, COL9A3 and COL6A5 expression in the metastatic nodules. Similar to the results of the western blot analyses, vimentin expression was suppressed after USP3 depletion; however, after COL9A3 or COL6A5 overexpression, vimentin was distinctly upregulated, suggesting that the EMT phenotype was rescued (Fig. 5D). The epithelial phenotype marker E-cadherin showed opposite results to above mesenchymal phenotype marker Vimentin. Overall, these data demonstrated that USP3 is involved in the EMT process to promote GC cell invasion and migration through COL9A3 and COL6A5.

### USP3 expression is positively associated with COL9A3 and COL6A5 expression, and the axis might be used as a prognosis biomarker for GC patients

**Fig. 6:**
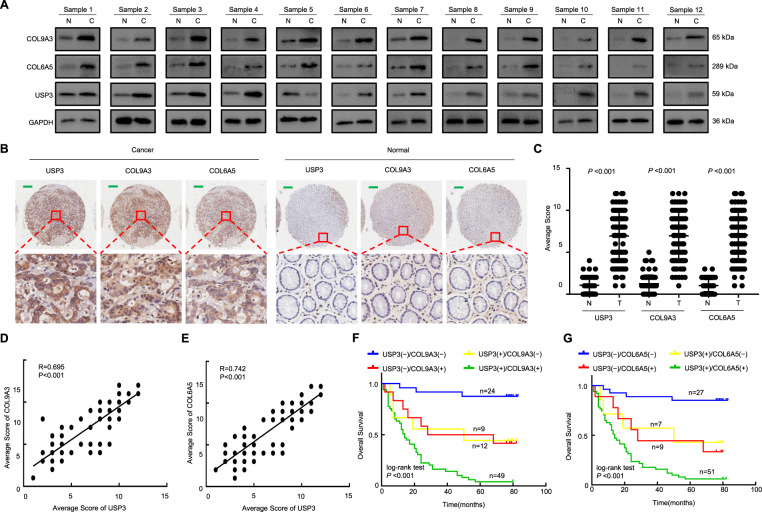
Positive association of USP3 expression with COL9A3 and COL6A5 expression, and the effect of the regulatory axis on prognosis of GC patients. **A** Western blot analysis of USP3, COL9A3 and COL6A5 levels in human GC tissues and adjacent normal tissues. **B** Representative images of USP3, COL9A3 and COL6A5 expressions in 94 samples of either normal or cancerous gastric tissues, which were detected by TMA analysis. Scale bars, 200 μm. **C** Scatter plot analysed the expression levels of USP3, COL9A3 and COL6A5 in GC tissues compared with the corresponding adjacent normal tissues. **D**, **E** Linear regression analysis and Pearson’s correlation were used to determine the relationship between USP3 and COL9A3/COL6A5 protein expression in gastric cancerous tissue specimens. **F**, **G** Kaplan-Meier curves for overall survival analysis of GC patients according to the associations between expression levels of USP3 and COL9A3/COL6A5.

We collected tumour tissues and corresponding normal tissues of gastric mucosa from 12 GC patients and detected the expression of USP3, COL9A3, and COL6A5 by western blotting (Fig. 6A). The results showed that USP3, COL9A3, and COL6A5 were highly expressed in GC tissues compared to normal tissues. Moreover, we further analysed USP3, COL9A3 and COL6A5 expression in tumour samples and adjacent normal tissues from 94 GC patients using TMA, along with their corresponding clinicopathologic information. Human GC tissues exhibited more intense immunostaining, whereas normal gastric tissues showed less (Fig. 6B). Semi-quantitative scoring showed that USP3, COL9A3 and COL6A5 proteins were expressed at significantly higher levels in cancer tissues than in adjacent normal tissues (Fig. 6C). Clinicopathological analysis revealed that high expression of COL9A3 and COL6A5 was correlated with poor tumour differentiation (*P* = 0.006 and 0.002, respectively), positive lymph node metastasis (both *P* < 0.001), large tumour size (both *P* < 0.001), advanced T stage (both *P* < 0.001), and late TNM stage (both *P* < 0.001). However, COL9A3/COL6A5 staining intensity was not associated with age (*P* = 0.913 and 0.613, respectively) or gender (*P* = 0.238 and 0.359, respectively) (Table 1).

**Table 1:** Correlation between COL9A3/COL6A5 protein expression and clinicopathologic characteristics of GC.

Characteristics	Total (*n* = 94)	COL9A3		*P* value	COL6A5		*P* value
		Low	High		Low	High	
Age (years)				0.913			0.613
<55	25	9	16		8	17	
≥55	69	24	45		26	43	
Gender				0.238			0.359
Male	39	11	28		12	27	
Female	55	22	33		22	33	
Differentiation				0.006			0.002
Well	28	16	12		17	11	
Moderate	33	11	22		11	22	
Poor	33	6	27		6	27	
Lymph node metastasis				<0.001			<0.001
Positive	71	16	55		17	54	
Negative	23	17	6		17	6	
Tumour size (cm^3^)				<0.001			<0.001
<10	23	19	4		19	4	
≥10	71	15	56		15	56	
AJCC T stage				<0.001			<0.001
T1, T2	14	12	2		12	2	
T3, T4	80	21	59		22	58	
Clinical TNM stage				<0.001			<0.001
I, II	38	26	12		26	12	
III, IV	56	7	49		8	48	

We also observed that higher expression of USP3 was associated with stronger expression of COL9A3 and COL6A5 in tumour tissues (Fig. 6B and D, E). Pearson’s correlation analyses revealed a significant positive correlation between the expression scores of USP3 and COL9A3 (*R* = 0.742, *P* < 0.001) (Fig. 6D), as well as between USP3 and COL6A5 (*R* = 0.695, *P* < 0.001) (Fig. 6E). We further explored the prognostic effect of such an expression association on survival outcomes of GC patients. The results showed that patients with high expression of either USP3 or COL9A3 had worse prognoses than those with low expression of both USP3 and COL9A3 (Fig. 6F). Notably, patients with simultaneous high expression of both USP3 and COL9A3 yielded the worst outcomes compared to those with only one or no high expression levels (Fig. 6F). Similar results were also found for USP and COL6A5 (Fig. 6G). These results suggest that the USP3-COL9A3/COL6A5 axis could serve as a prognostic marker and regulatory target for GC patients.

## Discussion

In a previous study, we have reported that USP3, as an important oncogene, is overexpressed in GC, promotes cell EMT, migration and invasion in vitro and in vivo, and is associated with poor prognosis in GC patients [14]. In this study, we further explored the biochemistry function for its inherent nature as a specific type of DUB, to understand the detailed mechanism between USP3 and malignant behaviours in GC, aiming to provide evidence for the complete USP3-involved regulatory network. We found that USP3 could interact with, deubiquitylate, and stabilise COL9A3 and COL6A5, thereby upregulating the protein expression of COL9A3 and COL6A5. Such deubiquitination-mediated overexpression of COL9A3 and COL6A5 could promote cell EMT, invasion, and migration in GC. High expression of COL9A3 or COL6A5 was an additional risk factor for USP3-modulated poor prognosis in GC patients. These findings demonstrate that deubiquitination-mediated overexpression of COL9A3/COL6A5 is an essential mediator of USP3 to promote GC progression. Moreover, the findings emphasise that the USP3-COL9A3/COL6A5 signalling axis plays a critical role in the development of GC, which could provide underlying therapeutic targets for GC management in clinical settings.

During tumour progression, excessive deposition of collagen can remodel the ECM, thereby enhancing the interaction between tumour cells and the microenvironment, resulting in tumour cell EMT and migration [22–24]. It has been reported that enriched collagen in the colorectal tumour microenvironment activates the PI3K/AKT signalling pathway via integrin α2β1, thus promoting tumour cell EMT and distant metastasis in colorectal cancer [25]. In breast cancer, the deposition of collagen activates LYN kinase to directly phosphorylate TWIST1 and drives tumour cell EMT and migration by increasing ECM stiffness [26]. In this study, we found that COL9A3 and COL6A5, the major components of type IX and VI collagen, respectively, were upregulated by USP3, which had been demonstrated in our previous work as a driver gene for EMT and metastasis in GC [14]. Here, our findings showed that overexpression of COL9A3 and COL6A5 caused distinct upregulation of the mesenchymal cell marker vimentin and downregulation of the epithelial cell marker E-cadherin, and reversed results were also observed after knockdown of COL9A3 or COL6A5. We have shown that an increase in USP3 expression causes EMT and cell migration in GC [14]. Interestingly, such events can be reversed by suppression of COL9A3 and COL6A5, suggesting that the regulation of the EMT process by USP3 is dependent on COL9A3 and COL6A5. These findings demonstrate that the alterations of components in the tumour microenvironment, such as collagens in the ECM, and their interaction with tumour cells, could provide new insights into USP3-mediated GC progression regarding EMT modulation.

We further explored the detailed interactions and regulatory mechanisms between USP3 and COL9A3 or COL6A5. As a DUB, USP3 has been identified to have deubiquitination effects on its wide targets within cellular entities and during cell activities in various malignancies [10, 11, 27, 28]. Das et al. have shown that USP3 can upregulate the expression of Cdc25A through its DUB activity, which promotes cell cycle progression and tumorigenesis [13]. Similarly, during the progression of glioblastomas, USP3 stabilises Snail via deubiquitination, resulting in tumour proliferation, invasion and migration [29]. However, to date, it is unclear whether USP3 upregulated its newly identified targets in the present study, i.e., COL9A3 and COL6A5, through its canonical deubiquitination function, as well as the detailed mechanism for such posttranslational modification. Our data in this study helped clarify this issue. Through the aforementioned experiments, we verified that there was an interaction between USP3 and COL9A3/COL6A5, and that USP3 could regulate the stability of COL9A3 and COL6A5 through its DUB function. The USP3 protein has been known to have two domains: ZnF and UCH [30]. A recent study has reported that the UCH domain can interact with Claspin and upregulate its expression, but not the ZnF domain [12]. Another study has also revealed that high expression of USP3-WT can stabilise Cdc25A protein levels in a dose-dependent manner, but not USP3-C168S, whose mutation causes the failure of UCH domain function in USP3 [13]. Here, we have identified that the UCH domain of USP3 can be combined with COL9A3/COL6A5 and that it plays a pivotal role in regulating stability, which further proves that the UCH domain in USP3 is crucial in exerting its deubiquitination function and regulating the stability of downstream molecules. The major deliverables for protein ubiquitination modification are the linkage of multiple ubiquitin chains with the protein substrate. Among these forms of ubiquitin chain, Lys 48- and 63-linked chains were observed as the most common types in humans [6, 31, 32]. The Lys 48-linked ubiquitin chain usually serves as an inducer of proteasome-mediated proteolysis, while the main function of Lys 63-linked ubiquitination is to regulate the activity of the protein. In the present study, our experimental data revealed that USP3 could regulate the stability of COL9A3 and COL6A5 by relieving their Lys 48-linked ubiquitination. Consistent results have also been found in several previous reports that USP3 removes the Lys 48-linked ubiquitin chain of downstream proteins to interrupt its degradation process and upregulate its protein expression level [12, 13]. Together, these findings help to fully elicit the relationship between the molecular conformation and function polymorphism of USP3. Moreover, it advances the completion of the USP3-mediated mechanism network, specifically in terms of deubiquitination modification.

Collagen forms the scaffold of the tumour microenvironment, which plays a vital role in tumour EMT, invasion, and metastasis [23, 33]. Some genes encoding collagen have been found to be aberrantly expressed during carcinogenesis in various types of cancers. For example, COL4A1, which encodes a major component of type IV collagen, acts as an oncogene to facilitate tumour cell metastasis via activation of the FAK-Src signalling pathway [34]. Some studies have reported that COL1A1 is upregulated in GC and can promote GC cell proliferation and invasion [35–37]. However, there are few reports on the relationship between COL9A3/COL6A5 and cancer. COL9A3 might be correlated with the pathogenesis of triple-negative breast cancer, and the polymorphisms of COL6A5 might be relevant to lung cancer susceptibility among Chinese Han individuals [38, 39]. Unfortunately, these previous results were obtained through in silico methods; hence, there is a lack of evidence from biological experiments to verify them. To our knowledge, this is the first study to detect the expression and function of COL9A3 and COL6A5 in GC. We found that COL9A3 and COL6A5 were highly expressed in GC tissues compared with adjacent normal tissues. In addition, COL9A3 and COL6A5 overexpression promoted EMT, migration and invasion of GC cells. Subsequent survival analysis suggested that high expression of COL9A3/COL6A5 predicted poor survival in GC patients. These findings indicate a potential relationship between COL9A3/COL6A5 expression and an aggressive phenotype in GC. Mechanistically, we initially explored the specific mechanism of COL9A3 and COL6A5 in EMT, invasion, and metastasis of GC cells from the perspective of ubiquitination modification. We found that deubiquitination modification was an effective regulator for maintaining the protein levels of COL9A3 and COL6A5, thereby establishing their foundation to drive GC cell EMT, invasion, and metastasis. Nonetheless, very little is known about the relationship between COL9A3 and COL6A5 and their downstream receptors as well as signalling pathways. Thus, further research is needed to provide systematic evidence to determine the roles and detailed mechanisms of COL9A3 and COL6A5, which also provide underlying targets for experimental design in the laboratory to clarify the complex mechanism of GC progression.

In conclusion, our study revealed a mechanism that accounted for the tumour-driver function of USP3 in GC progression. USP3 promoted GC cell migration, invasion, and EMT by interacting with and deubiquitinating COL9A3 and COL6A5. This study suggests that targeting of the USP3-COL9A3 or USP3-COL6A5 axis could be developed as a therapeutic strategy for GC management.

## Materials and methods

### Cell culture, antibodies and reagents

GC cells (AGS, HGC-27, BGC-823, HGC-27, and MGC-803) and human gastric mucosal epithelial cells (GES-1) were purchased from Beijing Institute of Cancer Research (Beijing, China). The cells were maintained in Dulbecco’s Modified Eagle Medium (DMEM) containing 10% foetal bovine serum (FBS; Invitrogen, Carlsbad, CA, USA) at 37 °C and 5% CO_2_.

Antibodies used in this experiment included anti-USP3 (12490-1-AP), anti-E-cadherin (20874-1-AP), anti-HA (51064-2-AP), anti-ubiquitin (10201-2-AP), anti-GAPDH (60004-1-Ig), anti-His (66005-1-Ig), anti-GST (10000-0-AP) and anti-vimentin (60330-1-Ig), all of which were purchased from Proteintech (Rosemont, IL, USA). Anti-COL9A3 (SAB1410224) was purchased from Sigma (Burlington, USA), Anti-COL6A5 (bs-11047R) from Bioss (Beijing, China), anti-Flag (14793) and anti-Myc (2276) antibodies from Cell Signaling Technology (Danvers, MA, USA), and MG132 and cycloheximide (CHX) from Selleck (Shanghai, China).

### Tissue microarray (TMA) and immunohistochemistry (IHC)

GC samples and the corresponding normal gastric mucosa tissues were used to construct a TMA (Outdo Biochip, Shanghai, China). Continuous TMAs were stained for USP3, COL9A3, and COL6A5 expression. The TMA was scored independently by two pathologists for both staining intensity and the extent of protein expression across the sections after immunohistochemical staining using the Dako Envision Plus System (Carpinteria, CA, USA), following the manufacturer’s instructions. The analysis was performed by two independent observers who were blinded to the clinical outcomes. Scoring of tissue slides was performed using a 12-tier scoring system in which the IHC score was calculated based on staining intensity and proportion of positive cells. The intensity of the cancer cell staining was scored as 0 for negative staining, 1 for weak staining (light yellow), 2 for moderate staining (yellow), and 3 for intense staining (yellowish brown); the proportion of positive cells was scored as 0 (1%–5%), 1 (6%–25%), 2 (26%–50%), 3(51%–75%) and 4 (76%–100%). The results were scored as previously described and as the product of the staining intensity and proportion of positive cells. Scores in the tumour area were evaluated in ten random fields, and the mean value was used to assess protein expression. Tissues with a final score of <5 were defined as low expressers, while those with ≥5 were defined as high expressers [40]. This study was approved by the institutional human ethics committee of the relevant institutions.

### Plasmid construction and small interfering RNA transfection

The COL9A3 or COL6A5 coding sequence was cloned into the pEZ shuttle plasmid (GeneCopoeia, Guangzhou, China). The empty vector, pEZ-COL9A3, and pEZ-COL6A5 plasmids encoding Myc or His tags were purchased from GeneCopoeia. Wild type and C168S mutant for USP3 were cloned into the pEZ-Flag vector (GeneCopoeia) to construct overexpression plasmids of full length USP3 and DUB-inactive USP3, respectively.

For RNA interference studies, GC cells were transfected with predesigned small interfering RNAs (siRNAs) at a final concentration of 25 nM. The siRNAs of USP3 sequences were as follows: siRNA1, TTCACAGCTGACAGGCATA; siRNA2, CCTTCAGTCACTCAGTAAC; and siRNA3, CCATGAATTCATGCGCTAC. The cells were transfected with plasmids or siRNA duplexes using Lipofectamine 3000 (Invitrogen Life Technologies, Carlsbad, CA, USA) according to the manufacturer’s instructions. The effect of translation was evaluated by western blotting 48 h post-transfection.

### Real-time qRT-PCR

Real-time qRT-PCR assays were performed as described previously [40]. To detect the mRNA expression of COL9A3 and COL6A5, real-time qRT-PCR was performed using SYBR Green (Yeasen Biotechnology, Shanghai, China). The forward and reverse PCR primers for COL9A3 were 5′-GTATTGCAGGTTCCGACGGTCT-3′ and 5′-TCTCCTTTGGGTCCGACCAG-3′, and for COL6A5 were 5′-AATCAGACGTGCCATCAACA-3′ and 5′-GGAGATGTTGTGCCTGGGAAT-3′. GAPDH was amplified as an internal control using the primers 5′-CACTGGGCTACACTGAGCAC-3′ and 5′-AGTGGTCGTTGAGGGCAAT-3′.

### Co-immunoprecipitation (Co-IP) and western blot analysis

Co-IP and western blot assays were performed as described previously [41]. To precipitate the target proteins, 1 mL of post-centrifuged lysates was incubated with 0.5–2.5 μg of antibody at 4 °C overnight, followed by incubation with a 50% slurry of protein A/G agarose (Santa Cruz Biotechnology, Texas, USA) for 2 h at 4 °C. The immunoprecipitated proteins were eluted with SDS-PAGE loading buffer by boiling for 5 min. For the western blot analysis, proteins were resolved by SDS-PAGE and transferred onto polyvinylidene difluoride membranes, followed by incubation with the corresponding antibodies overnight at 4 °C. The next day, membranes were incubated with their respective secondary antibodies, washed three times with TBST and visualised using chemiluminescence.

### Isobaric tags for relative and absolute quantitation (iTRAQ) and pathway enrichment analysis

Cells were collected and transferred to the Beijing Genomics Institute (Shenzhen, China) for further treatment and analysis, including protein preparation, iTRAQ labelling, 2D LC-MS/MS, data processing, and database searching, which were performed as previously reported [14]. The false discovery rate was set to 0.01 for both peptide and protein identification. Finally, differentially expressed proteins were defined with a 1.2-fold change (mean value of all comparison groups) and *P* value (*t* test of all comparison groups) less than 0.05. The differentially expressed genes were used for KEGG pathway enrichment analysis using the WEB-based GEne SeT AnaLysis Toolkit (WebGestalt, http://www.webgestalt.org/).

### Wound-healing assay

The cells were seeded in six-well plates and cultured overnight. The confluent monolayers were wounded in a line across the slides with a sterile 20 μL plastic pipette tip. The wound was then washed twice with PBS. Cells were further cultured in a medium containing 2% FBS for 48 h. The distances migrated by the cell monolayer to close the wound area during the indicated time were measured. Cell migration, indicating wound-healing effect, is expressed as a migration index, i.e., the distance migrated by GC cells at the indicated point in time relative to the initial length of the wound. Experiments were performed at least in at least triplicate.

### Invasion assay

After the cells were resuspended in serum-free DMEM, a 200 μL cell suspension (1 × 10^5^ cells) was added to the upper compartment of migration chambers coated with Matrigel (BD Biosciences, Boston, MA, USA). The bottom chamber was filled with 500 μL of DMEM with 10% FBS. After 48 h, the cells were fixed with 4% paraformaldehyde for 40 min and stained with 5% crystal violet for 30 min. The number of invading cells was counted in five random visual fields per chamber under a microscope (Olympus, Tokyo, Japan). Experiments were performed at least in triplicate.

### Immunofluorescence staining and confocal laser scanning microscopy

Cells were plated onto sterilised glass coverslips, washed in PBS, fixed with 4% paraformaldehyde for 10 min, and permeabilized with 0.25% Triton for 5 min. The slides were then blocked in PBS plus 5% FBS at 37 °C for 1 h, followed by incubation with antibodies at 4 °C overnight. The next day, the cells were incubated with fluorescently conjugated secondary antibodies for 1 h and imaged using a confocal laser scanning microscope (Olympus, Center Valley, PA, USA).

### Protein half-life assay

Cells were transfected with the corresponding plasmid or siRNA and were consequently treated with 100 μM CHX for the times indicated in the figure legends. At the indicated time points, cells were collected and analysed by western blotting to determine the abundance of endogenous COL9A3 or COL6A5 proteins.

### Deubiquitination assay

Deubiquitination assays were conducted as described previously [14]. In brief, to analyse the ubiquitination of COL9A3 or COL6A5, cells were co-transfected with HA-Ub and Flag-USP3 (or vector) for 48 h. After the cells were treated with 20 μM of the proteasome inhibitor MG132 for 8 h, cell lysates were prepared with cell lysis buffer and immunoprecipitated with the indicated antibodies (anti-COL9A3, anti-COL6A5) on protein A/G beads (Santa Cruz Biotechnology, Texas, USA) overnight. The beads were then washed and boiled in SDS loading buffer. Immunoprecipitated protein complexes were assessed by western blotting with anti-HA.

### Construction of lentivirus and mouse metastatic model

The shRNA of USP3 was designed and synthesised according to siRNA2 by Genechem Co. LTD. Lentiviruses expressing COL9A3 (LV-COL9A3) and COL6A5 (LV-COL6A5) were constructed by Genechem (Shanghai, China) using the Ubi-MCS-SV40-Cherry-IRES-neomycin vector; an empty vector was used as a control. To establish USP3 knockdown and COL9A3/COL6A5 overexpression (USP3-shRNA + COL9A3 or COL6A5) cell lines, lentivirus-expressing COL9A3/COL6A5 cDNA, and USP3 shRNA were co-transfected into the GC cell lines. After 72 h, puromycin or G418 was used for the selection of stable clones.

The tail-vein model which results in lung metastasis by human GC cells, was established as described previously [42]. A single-cell suspension of 5 × 10^6^ BGC-823 Scr-shRNA + Vector, USP3-shRNA + Vector, USP3-shRNA + COL9A3, or USP3-shRNA + COL6A5 transduced cells/100 μl PBS was injected via the tail vein. The progression of cancer cell growth was monitored after 34 days by bioluminescent imaging using the IVIS100 imaging system (Kodak, Rochester, NY, USA). The metastatic tissues were analysed using HE and IHC staining. The Committee on the Use of Live Animals in Teaching and Research (Southern Medical University, China) approved the protocol.

### Statistical analysis

Statistical analyses were performed using SPSS (version 20.0, Chicago, IL, USA). Quantitative data obtained from experiments with biological replicates are shown as mean ± standard deviation. Linear regression and Pearson correlation coefficients were employed to evaluate the correlation. Survival curves were plotted using the Kaplan–Meier estimator and compared using log-rank tests. Student’s *t* test was used to analyse the quantitative data, while the Chi-square test (or Fisher’s exact test, if appropriate) was used to analyse categorical data. A two-tailed *P* value of <0.05, was considered statistically significant.

## Supplementary information

Supplementary Figure 1

Supplementary Figure 2

Supplementary Figure 3

Supplementary Figure 4

Supplementary Figure Legends

Checklist

Change of Authorship Request Form

Certificate_of language polishing

Author contribution form

## Data Availability

All remaining data are available within the article and supplementary files and are available from the authors upon request.

## References

[1] Sung H, Ferlay J, Siegel RL, Laversanne M, Soerjomataram I, Jemal A (2021). Global Cancer Statistics 2020: GLOBOCAN estimates of incidence and mortality worldwide for 36 cancers in 185 countries. CA Cancer J Clin.

[2] Yu J, Huang C, Sun Y, Su X, Cao H, Hu J (2019). Effect of laparoscopic vs open distal gastrectomy on 3-year disease-free survival in patients with locally advanced gastric cancer: the CLASS-01 randomized clinical trial. JAMA..

[3] Smyth EC, Nilsson M, Grabsch HI, van Grieken NC, Lordick F (2020). Gastric cancer. Lancet..

[4] Wei W, Zeng H, Zheng R, Zhang S, An L, Chen R (2020). Cancer registration in China and its role in cancer prevention and control. Lancet Oncol.

[5] Carroll EC, Greene ER, Martin A, Marqusee S (2020). Site-specific ubiquitination affects protein energetics and proteasomal degradation. Nat Chem Biol.

[6] Swatek KN, Usher JL, Kueck AF, Gladkova C, Mevissen TET, Pruneda JN (2019). Insights into ubiquitin chain architecture using Ub-clipping. Nature..

[7] Mevissen TET, Komander D (2017). Mechanisms of deubiquitinase specificity and regulation. Annu Rev Biochem.

[8] Celebi G, Kesim H, Ozer E, Kutlu O (2020). The effect of dysfunctional ubiquitin enzymes in the pathogenesis of most common diseases. Int J Mol Sci..

[9] Harrigan JA, Jacq X, Martin NM, Jackson SP (2018). Deubiquitylating enzymes and drug discovery: emerging opportunities. Nat Rev Drug Discov.

[10] Wu Y, Qin J, Li F, Yang C, Li Z, Zhou Z (2019). USP3 promotes breast cancer cell proliferation by deubiquitinating KLF5. J Biol Chem.

[11] Liao XH, Wang Y, Zhong B, Zhu SY (2020). USP3 promotes proliferation of non-small cell lung cancer through regulating RBM4. Eur Rev Med Pharm Sci.

[12] Tu Y, Chen Z, Zhao P, Sun G, Bao Z, Chao H (2020). Smoothened promotes glioblastoma radiation resistance via activating USP3-mediated claspin deubiquitination. Clin Cancer Res.

[13] Das S, Chandrasekaran AP, Suresh B, Haq S, Kang JH, Lee SJ (2020). Genome-scale screening of deubiquitinase subfamily identifies USP3 as a stabilizer of Cdc25A regulating cell cycle in cancer. Cell Death Differ.

[14] Wu X, Liu M, Zhu H, Wang J, Dai W, Li J (2019). Ubiquitin-specific protease 3 promotes cell migration and invasion by interacting with and deubiquitinating SUZ12 in gastric cancer. J Exp Clin Cancer Res.

[15] Fang CL, Lin CC, Chen HK, Hseu YC, Hung ST, Sun DP (2018). Ubiquitin-specific protease 3 overexpression promotes gastric carcinogenesis and is predictive of poor patient prognosis. Cancer Sci.

[16] Xu S, Xu H, Wang W, Li S, Li H, Li T (2019). The role of collagen in cancer: from bench to bedside. J Transl Med.

[17] Martins Cavaco AC, Damaso S, Casimiro S, Costa L (2020). Collagen biology making inroads into prognosis and treatment of cancer progression and metastasis. Cancer Metastasis Rev.

[18] Zhu H, Chen H, Wang J, Zhou L, Liu S (2019). Collagen stiffness promoted non-muscle-invasive bladder cancer progression to muscle-invasive bladder cancer. Onco Targets Ther.

[19] Jin H, He Y, Zhao P, Hu Y, Tao J, Chen J (2019). Targeting lipid metabolism to overcome EMT-associated drug resistance via integrin beta3/FAK pathway and tumor-associated macrophage repolarization using legumain-activatable delivery. Theranostics..

[20] Izdebska M, Zielinska W, Halas-Wisniewska M, Grzanka A (2020). Involvement of actin and actin-binding proteins in carcinogenesis. Cells.

[21] Pastushenko I, Blanpain C (2019). EMT transition states during tumor progression and metastasis. Trends Cell Biol.

[22] Emon B, Bauer J, Jain Y, Jung B, Saif T (2018). Biophysics of tumor microenvironment and cancer metastasis—a mini review. Comput Struct Biotechnol J..

[23] Paolillo M, Schinelli S. Extracellular matrix alterations in metastatic processes. Int J Mol Sci. 2019;20:4947. 10.3390/ijms20194947 PMC6802000 31591367

[24] Guan X (2015). Cancer metastases: challenges and opportunities. Acta Pharm Sin B..

[25] Wu X, Cai J, Zuo Z, Li J (2019). Collagen facilitates the colorectal cancer stemness and metastasis through an integrin/PI3K/AKT/Snail signaling pathway. Biomed Pharmacother.

[26] Fattet L, Jung HY, Matsumoto MW, Aubol BE, Kumar A, Adams JA (2020). Matrix rigidity controls epithelial-mesenchymal plasticity and tumor metastasis via a mechanoresponsive EPHA2/LYN complex. Dev Cell.

[27] Chen W, Li Q, Zhang G, Wang H, Zhu Z, Chen L (2020). LncRNA HOXA-AS3 promotes the malignancy of glioblastoma through regulating miR-455-5p/USP3 axis. J Cell Mol Med.

[28] Nagy Z, Seneviratne JA, Kanikevich M, Chang W, Mayoh C, Venkat P (2021). An ALYREF-MYCN coactivator complex drives neuroblastoma tumorigenesis through effects on USP3 and MYCN stability. Nat Commun.

[29] Fan L, Chen Z, Wu X, Cai X, Feng S, Lu J (2019). Ubiquitin-specific protease 3 promotes glioblastoma cell invasion and epithelial-mesenchymal transition via stabilizing snail. Mol Cancer Res.

[30] Nicassio F, Corrado N, Vissers JH, Areces LB, Bergink S, Marteijn JA (2007). Human USP3 is a chromatin modifier required for S phase progression and genome stability. Curr Biol.

[31] Akutsu M, Dikic I, Bremm A (2016). Ubiquitin chain diversity at a glance. J Cell Sci.

[32] Swatek KN, Komander D (2016). Ubiquitin modifications. Cell Res.

[33] Druzhkova I, Shirmanova M, Ignatova N, Dudenkova V, Lukina M, Zagaynova E, et al. Expression of EMT-related genes in hybrid E/M colorectal cancer cells determines fibroblast activation and collagen remodeling. Int J Mol Sci. 2020;21:8119. 10.3390/ijms21218119 PMC7662237 33143259

[34] Wang T, Jin H, Hu J, Li X, Ruan H, Xu H (2020). COL4A1 promotes the growth and metastasis of hepatocellular carcinoma cells by activating FAK-Src signaling. J Exp Clin Cancer Res.

[35] Guo Y, Lu G, Mao H, Zhou S, Tong X, Wu J (2020). miR-133b suppresses invasion and migration of gastric cancer cells via the COL1A1/TGF-beta axis. Onco Targets Ther.

[36] Wang Q, Yu J (2018). MiR-129-5p suppresses gastric cancer cell invasion and proliferation by inhibiting COL1A1. Biochem Cell Biol.

[37] Shi Y, Duan Z, Zhang X, Zhang X, Wang G, Li F (2019). Down-regulation of the let-7i facilitates gastric cancer invasion and metastasis by targeting COL1A1. Protein Cell.

[38] Lv X, He M, Zhao Y, Zhang L, Zhu W, Jiang L (2019). Identification of potential key genes and pathways predicting pathogenesis and prognosis for triple-negative breast cancer. Cancer Cell Int.

[39] Duan Y, Liu G, Sun Y, Wu J, Xiong Z, Jin T (2020). Collagen type VI alpha5 gene variations may predict the risk of lung cancer development in Chinese Han population. Sci Rep.

[40] Wang J, Wu X, Dai W, Li J, Xiang L, Tang W (2020). The CCDC43-ADRM1 axis regulated by YY1, promotes proliferation and metastasis of gastric cancer. Cancer Lett.

[41] Zhang H, Wu X, Xiao Y, Wu L, Peng Y, Tang W (2019). Coexpression of FOXK1 and vimentin promotes EMT, migration, and invasion in gastric cancer cells. J Mol Med.

[42] Zhu H, Dai W, Li J, Xiang L, Wu X, Tang W (2019). HOXD9 promotes the growth, invasion and metastasis of gastric cancer cells by transcriptional activation of RUFY3. J Exp Clin Cancer Res.

