# Supplementary material for: USP3 promotes gastric cancer progression and metastasis by deubiquitination-dependent COL9A3/COL6A5 stabilisation
Source: Cell Death Dis. 2021 Dec 20;13(1):10. doi: 10.1038/s41419-021-04460-7 (PMC8688524; doi:10.1038/s41419-021-04460-7)
Supplement: Supplementary file 5 — Supplementary Figure Legends [file 41419_2021_4460_MOESM5_ESM.docx]

**Supplemental Figure 1. USP3 stabilizes COL9A3 and COL6A5 proteins. A.** Real time qRT-PCR detected the COL9A3 and COL6A5 mRNA levels in GC cells with overexpression of USP3 or the knock down of USP3. **B.** AGS and BGC-823 cells transfected with Scr-siRNA or USP3-siRNA1 were treated with cycloheximide (10 μg·ml^-1^), and collected at the indicated times for western blot. Quantifications of COL9A3 and COL6A5 levels are shown.

**Supplemental Figure 2. USP3 upregulates COL9A3 and COL6A5 to promote GC cells invasion and migration *in vitro*. A & C.** Statistical analysis for the number of cells passing through the chamber in the transwell assays. **B & D.** Cells were analyzed with live-cell microscopy in the wound-healing experiments. ***, *P* <0.001.

**Supplemental Figure 3. COL9A3 and COL6A5 are involved in USP3-induced GC cell EMT, invasion and migration.** COL9A3 or COL6A5 siRNA was introduced into the USP3 plasmid-transduced AGS and BGC-823 cells. **A & B.** E-cadherin and Vimentin were analyzed by western blot and immunofluorescence. Scale bars, 20 μm. **C & D.** GC cells invasion and migration assays were performed. The error bars represent the mean ± SD from 3 independent experiments.

**Supplemental Figure 4. COL9A3 and COL6A5 are involved in USP3-induced GC cell EMT, invasion and migration.** USP3 plasmid was introduced into the COL9A3 or COL6A5 knocked down AGS and BGC-823 cells. **A.** USP3, COL9A3 and COL6A5 were analyzed by western blot. **B, C, D &E.** GC cells invasion and migration assays were performed. The error bars represent the mean ± SD from 3 independent experiments.
