# Supplementary material for: USP3 promotes gastric cancer progression and metastasis by deubiquitination-dependent COL9A3/COL6A5 stabilisation
Source: Cell Death Dis. 2021 Dec 20;13(1):10. doi: 10.1038/s41419-021-04460-7 (PMC8688524; doi:10.1038/s41419-021-04460-7)
Supplement: Supplementary file 7 — Change of Authorship Request Form [file 41419_2021_4460_MOESM7_ESM.pdf]

SPRINGER NATURE

Change of authorship request form - Journals (pre-acceptance)

Important information. Please read.

- This form should be used by authors to request any change in authorship (adding/deleting authors) including changes in corresponding authors. This form should not be used for name changes. Please fully complete all sections. Use black ink and block capitals and provide each author's full name with the given name first followed by the family name.

- By signing this declaration, all authors guarantee that the order of the authors are in accordance with their scientific contribution, if applicable as different conventions apply per discipline, and that only authors have been added who made a meaningful contribution to the work.

- Please note, in author collaborations where there is formal agreement for representing the collaboration, it is sufficient for the representative or legal guarantor (usually the corresponding author) to complete and sign the Authorship Change Form on behalf of all authors, **next to the added/removed author(s)**. **(Complete Section 3, followed by Section 6.)**
  In author collaborations where there is no formal agreement for representing the collaboration and **there are more than 10 authors**, one may sign for all, provided the signer appends correspondence that attests that each of the authors have agreed to the change **and the added/removed authors sign the form**. **(Complete Section 3, followed by Section 6.)**

- Please note, we cannot investigate or mediate any authorship disputes. If you are unable to obtain agreement from all authors (including those who you wish to be removed) you must refer the matter to your institution(s) for investigation. Please inform us if you need to do this.

- If you are not able to return a fully completed form within **30 days** of the date that it was sent to the author requesting the change, we may have to withdraw your manuscript. We cannot publish manuscripts where authorship has not been agreed by all authors (including those who have been removed).

- Incomplete forms will be rejected.

- Please return/upload this form, fully completed, to the Journals Editorial Office. The Journal and/or Publisher will consider the information you have provided to decide whether to approve the proposed change in authorship. We may decide to contact your institution for more information or undertake a further investigation, if appropriate, before making a final decision.

SPRINGER NATURE Springer Nature is one of the world's leading global research, educational and professional publishers, created in May 2015 through the combination of Nature Publishing Group, Palgrave Macmillan, Macmillan Education and Springer Science+Business Media.

SPRINGER NATURE

Change of authorship request form - Journals (pre-acceptance)

Section 1: Please provide the current title of manuscript

Manuscript ID no.: CDDIS-21-2560R

Title: USP3 promotes gastric cancer progression and metastasis by deubiquitination-dependent COL9A3/COL6A5 stabilisation

Section 2: Please provide the previous authorship, in the order shown on the manuscript before the changes were introduced. Please indicate the corresponding author by adding (CA) behind the name.

	First name(s)	Family name	ORCID or SCOPUS id, if available
1st author	Xiaosheng	Wu	
2nd author	Hao	Wang	
3rd author	Danping	Zhu	
4th author	Jing	Wang	
5th author	Weiyu	Dai	
6th author	Yizhi	Xiao	
7th author	Weimei	Tang	
8th author	Jiaying	Li	
9th author	Linjie	Hong	
10th author	Miaomiao	Pei	
11th author	Jieming	Zhang	
12th author	Zhizhao	Lin	
13th author	Jide	Wang	
14th author	Aimin	Li	
15th author	Side	Liu	

Please use an additional sheet if there are more than 10 authors.

SPRINGER NATURE Springer Nature is one of the world's leading global research, educational and professional publishers, created in May 2015 through the combination of Nature Publishing Group, Palgrave Macmillan, Macmillan Education and Springer Science+Business Media.

Change of authorship request form - Journals (pre-acceptance)

Section 3: Please provide a justification for change. Please use this section to explain your reasons for changing the authorship of your manuscript, e.g. what necessitated the change in authorship? Please refer to the (journal) policy pages for more information about authorship. Please explain why omitted authors were not originally included and/or why authors were removed on the submitted manuscript.

Dear Dr. Peschiaroli,

We are deeply sorry to make a change in the order of the authors. We wish to add Dr. Yixia Chai to the fourth place. The reasons are that in the process of this article revision, Dr. Yixia Chai have make significant efforts on completing the experiment that needs to be supplemented and modified. We awfully apologize for this inconvenience and hope that this change is acceptable. However, please advise if you have any questions about this change. All authors have agreed with this change and declared no conflict of interest.

Thank you in advance for your understanding and kind assistance, and look forward to receiving your advice soon.

Sincerely,

Side Liu PhD, Aimin Li PhD & Jide Wang PhD
Department of Digestive Medicine, Nanfang Hospital,
Southern Medical University, Guangzhou, 510515,
Guangdong Province, China
Tel: 86-020-61645169
Email: liuside2011@163.com, lam0725@163.com, jidewang55@163.com

---
SPRINGER NATURE Springer Nature is one of the world's leading global research, educational and professional publishers, created in May 2015 through the combination of Nature Publishing Group, Palgrave Macmillan, Macmillan Education and Springer Science+Business Media.

SPRINGER NATURE

Change of authorship request form - Journals (pre-acceptance)

Section 4: Proposed new authorship. Please provide your new authorship list in the order you would like it to appear on the manuscript. Please indicate the corresponding author by adding (CA) behind the name. <u>If the Corresponding Author has changed, please indicate the reason under section 3</u>.

	First name(s)	Family name (this name will appear in full on the final publication and will be searchable in various abstract and indexing databases)	Affiliated institute	E-mail address
1st author	Xiaosheng	Wu	Nanfang Hospital, Southern Medical	
2nd author	Hao	Wang	Nanfang Hospital, Southern Medical	
3rd author	Danping	Zhu	Rocket Army Guangzhou Special Service	
4th author	Yixia	Chai	Nanfang Hospital, Southern Medical	
5th author	Jing	Wang	School of Basic Medical Sciences, Southern	
6th author	Weiyu	Dai	Nanfang Hospital, Southern Medical	
7th author	Yizhi	Xiao	Nanfang Hospital, Southern Medical	
8th author	Weimei	Tang	Nanfang Hospital, Southern Medical	
9th author	Jiaying	Li	Nanfang Hospital, Southern Medical	
10th author	Linjie	Hong	Nanfang Hospital, Southern Medical	
11th author	Miaomiao	Pei	Nanfang Hospital, Southern Medical	
12th author	Jieming	Zhang	Nanfang Hospital, Southern Medical	
13th author	Zhizhao	Lin	Nanfang Hospital, Southern Medical	
14th author	Jide	Wang	Nanfang Hospital, Southern Medical	
15th author	Aimin	Li	Nanfang Hospital, Southern Medical	
16th author	Side	Liu	Nanfang Hospital, Southern Medical	

Please use an additional sheet if there are more than 10 authors.

SPRINGER NATURE Springer Nature is one of the world's leading global research, educational and professional publishers, created in May 2015 through the combination of Nature Publishing Group, Palgrave Macmillan, Macmillan Education and Springer Science+Business Media.

Change of authorship request form - Journals (pre-acceptance)

Section 5: Author contribution, Acknowledgement and Disclosures. Please use this section to provide a new disclosure statement and, if appropriate, acknowledge any contributors who have been removed as authors and ensure you state what contribution any new authors made (if applicable per the journal or book (series) policy). Please ensure these are updated in your manuscript - after approval of the change(s) - as our production department will not transfer the information in this form to your manuscript.

New acknowledgements:

We acknowledge the generous support of the Guangdong Provincial Key Laboratory of Gastroenterology, Department of Gastroenterology, Nanfang Hospital of Southern Medical University.

New Disclosures (financial and non-financial interests, funding):

This study was supported by grants from the National Natural Science Funds of China (Nos. 82103598, 81772964, 81974448, and 82073066), President Foundation of Nanfang Hospital, Southern Medical University (No. 2020C005), Hainan Provincial Natural Science Foundation of China (No. 820QN390), Guangdong Gastrointestinal Disease Research Center (No. 2017B020209003), the Special Scientific Research Fund of Public Welfare Profession of National Health and Family Planning Commission (No. 201502026), Guangdong Medical Research Foundation (No. B2019126), and Shenzhen Science and Technology Innovation Commission (No. JCYJ20180306170328854).

New Author Contributions statement (if applicable per the journal policy):

This study was designed and conceived by SL, AL, and JW. *In vitro* experiments were performed by YC, XW, HW, DZ, and JW. *In vivo* experiments were conducted by XW, WD, YX, WT, and JL. LH, YC, and MP helped with data analysis. JZ and ZL were responsible for specimen collection. ZL contributed to technical support. SL, AL, and JW supervised the project. XW and HW drafted the manuscript. All authors read and approved the final manuscript.

State 'Not applicable' if there are no new authors.

SPRINGER NATURE Springer Nature is one of the world's leading global research, educational and professional publishers, created in May 2015 through the combination of Nature Publishing Group, Palgrave Macmillan, Macmillan Education and Springer Science+Business Media.

SPRINGER NATURE **Change of authorship request form - Journals** (pre-acceptance)

Section 6: Declaration of agreement. *All* authors, unchanged, new and removed *must* sign this declaration.
(NB: Please print the form, (docu)-sign and return/upload a scanned copy. Please note that signatures that have been inserted as an image file are acceptable as long as it is handwritten. Typed names in the signature box are unacceptable.) *** Please delete as appropriate. Delete all of the bold if you were on the original authorship list and are remaining as an author.**

	First name	Family name		Signature	Date
1st author	Xiaosheng	Wu	I agree to the proposed new authorship shown in section 4 **/and the addition/removal*of my name to the authorship list** /and the proposed change in corresponding author	Xiaosheng Wu	October 27, 2021
2nd author	Hao	Wang	I agree to the proposed new authorship shown in section 4 **/and the addition/removal*of my name to the authorship list** /and the proposed change in corresponding author	Hao Wang	October 27, 2021
3rd author	Danping	Zhu	I agree to the proposed new authorship shown in section 4 **/and the addition/removal*of my name to the authorship list** /and the proposed change in corresponding author	Danping Zhu	October 27, 2021
4th authors	Yixia	Chai	I agree to the proposed new authorship shown in section 4 **/and the addition/removal*of my name to the authorship list** /and the proposed change in corresponding author	Yixia Chai	October 27, 2021
5th author	Jing	Wang	I agree to the proposed new authorship shown in section 4 **/and the addition/removal*of my name to the authorship list** /and the proposed change in corresponding author	Jing Wang	October 27, 2021
6th author	Weiyu	Dai	I agree to the proposed new authorship shown in section 4 **/and the addition/removal*of my name to the authorship list** /and the proposed change in corresponding author	Weiyu Dai	October 27, 2021
7th author	Yizhi	Xiao	I agree to the proposed new authorship shown in section 4 **/and the addition/removal*of my name to the authorship list** /and the proposed change in corresponding author	Yizhi Xiao	October 27, 2021

SPRINGER NATURE Springer Nature is one of the world's leading global research, educational and professional publishers, created in May 2015 through the combination of Nature Publishing Group, Palgrave Macmillan, Macmillan Education and Springer Science+Business Media.

8th author	Weimei	Tang	I agree to the proposed new authorship shown in section 4 **/and the addition/removal*of my name to the authorship list** /and the proposed change in corresponding author	Weimei Tang	October 27, 2021
9th author	Jiaying	Li	I agree to the proposed new authorship shown in section 4 **/and the addition/removal*of my name to the authorship list** /and the proposed change in corresponding author	Jiaying Li	October 27, 2021
10th author	Linjie	Hong	I agree to the proposed new authorship shown in section 4 **/and the addition/removal*of my name to the authorship list** /and the proposed change in corresponding author	Linjie Hong	October 27, 2021
11th author	Miaomiao	Pei	I agree to the proposed new authorship shown in section 4 **/and the addition/removal*of my name to the authorship list** /and the proposed change in corresponding author	Miaomiao Pei	October 27, 2021
12th author	Jieming	Zhang	I agree to the proposed new authorship shown in section 4 **/and the addition/removal*of my name to the authorship list** /and the proposed change in corresponding author	Jieming Zhang	October 27, 2021
13th author	Zhizhao	Lin	I agree to the proposed new authorship shown in section 4 **/and the addition/removal*of my name to the authorship list** /and the proposed change in corresponding author	Zhizhao Lin	October 27, 2021
14th author	Jide	Wang	I agree to the proposed new authorship shown in section 4 **/and the addition/removal*of my name to the authorship list** /and the proposed change in corresponding author	Jide Wang	October 27, 2021
15th author	Aimin	Li	I agree to the proposed new authorship shown in section 4 **/and the addition/removal*of my name to the authorship list** /and the proposed change in corresponding author	Aimin Li	October 27, 2021
16th author	Side	Liu	I agree to the proposed new authorship shown in section 4 **/and the addition/removal*of my name to the authorship list** /and the proposed change in corresponding author	Side Liu	October 27, 2021

SPRINGER NATURE

Change of authorship request form - Journals (pre-acceptance)

Please use an additional sheet if there are more than 10 authors.

In case of author collaborations with formal agreement:

	Name of consortium/consorti	First name	Family name		Signature	Date
Representative/legal guarantor				I agree to the proposed new authorship shown in section 4 /**and the addition/removal*of my name to the authorship list** /and the proposed change in corresponding author		

Both added/removed authors should complete the information in the first table under Section 6.

---- End of form ----

SPRINGER NATURE Springer Nature is one of the world's leading global research, educational and professional publishers, created in May 2015 through the combination of Nature Publishing Group, Palgrave Macmillan, Macmillan Education and Springer Science+Business Media.
