# Supplementary material for: USP3 promotes gastric cancer progression and metastasis by deubiquitination-dependent COL9A3/COL6A5 stabilisation
Source: Cell Death Dis. 2021 Dec 20;13(1):10. doi: 10.1038/s41419-021-04460-7 (PMC8688524; doi:10.1038/s41419-021-04460-7)
Supplement: Supplementary file 9 — Author contribution form [file 41419_2021_4460_MOESM9_ESM.pdf]

DECLARATION OF CONTRIBUTIONS TO ARTICLE

ADMC

Manuscript Number: CDDIS-21-2560

Journal Name: *Cell Death & Disease* (the 'Journal')

Proposed Title of the Contribution:

USP3 promotes gastric cancer progression and metastasis by deubiquitination-dependent COL9A3/COL6A5 stabilization (the 'Contribution')

Author(s):

Xiaosheng Wu, Hao Wang, Danping Zhu, Jing Wang, Weiyu Dai, Yizhi Xiao, Weimei Tang, Jiaying Li, Linjie Hong, Miaomiao Pei, Jieming Zhang1 Zhizhao Lin, Jide Wang, Aimin Li, Side Liu (the 'Authors')

For all *CDDis* articles, each person named as an author in the published version must be able to show he or she has contributed substantially to the article.

Authorship credit should be based on 1) substantial contributions to conception and design, acquisition of data, or analysis and interpretation of data; 2) drafting the article or revising it critically for important intellectual content; and 3) final approval of the version to be published. Authors should meet conditions 1, 2 and 3.

Any person who cannot be shown to have made a substantial contribution to the article cannot be listed as an author in the final version. The name of any person who is deemed to have made a minor contribution can, however, appear in the Acknowledgments section of the article.

Please complete the table below to indicate the contributions of all named authors to the manuscript.

Author Full Name:	Specification of Contribution to the Manuscript:
Side Liu, Aimin Li and Jide Wang	Design of this study; supervision of this project; revision of the manuscript.
Xiaosheng Wu, Hao Wang, Danping Zhu and Jing Wang	Experiments in vitro; revision of the manuscript.
Xiaosheng Wu, Weiyu Dai, Yizhi Xiao, Weimei Tang and Jiaying Li	Experiments in vivo; revision of the manuscript.
Linjie Hong and Miaomiao Pei	Data analysis; revision of the manuscript.
Jieming Zhang and Zhizhao Lin	Collection of specimens; revision of the manuscript.
Xiaosheng Wu and Hao Wang	Manuscript draft; revision of the manuscript.
All authors	Approval of the final vision of the manuscript.

Please complete the table below to indicate the contributions of all named authors to the figures.

Figure 1:

Xiaosheng Wu, Hao Wang, Jing Wang and Miaomiao Pei

Figure 2:

Xiaosheng Wu, Hao Wang and Danping Zhu

Figure 3:

Xiaosheng Wu, Danping Zhu and Jing Wang

Figure 4:

Xiaosheng Wu, Hao Wang, Danping Zhu, Jing Wang and Linjie Hong

Figure 5:

Xiaosheng Wu, Weiyu Dai, Yizhi Xiao, Weimei Tang, Jiaying Li, Linjie Hong and Miaomiao Pei

Figure 6:

Xiaosheng Wu, Hao Wang, Danping Zhu, Jing Wang, Jieming Zhang, Zhizhao Lin, Linjie Hong and Miaomiao Pei

Signed for and on behalf of the Author(s):	Print Name:	Date:
Side Liu	Side Liu	2021-06-30
